# A novel mouse model for familial hypocalciuric hypercalcemia (FHH1) reveals PTH-dependent and independent CaSR defects

**DOI:** 10.1007/s00424-024-02927-y

**Published:** 2024-02-22

**Authors:** Catharina J. Küng, Arezoo Daryadel, Rocio Fuente, Betül Haykir, Martin Hrabĕ de Angelis, Nati Hernando, Isabel Rubio-Aliaga, Carsten A. Wagner

**Affiliations:** 1 Institute of Physiology, University of Zürich, Winterthurerstrasse 190, CH-8057 Zurich, Switzerland; 2 Department of Morphology and Cellular Biology, University of Oviedo, Oviedo, Spain; 3 Institute of Experimental Genetics, Helmholtz Zentrum München, German Research Center for Environmental Health, Neuherberg, Germany; 4 Lehrstuhl Für Experimentelle Genetik, Technische Universität München, Freising-Weihenstephan, Germany; 5 Member of German Center for Diabetes Research (DZD), Neuherberg, Germany

**Keywords:** Calcium-sensing receptor, Kidney, Bone, Parathyroid hormone, Fibroblast growth factor 23, Mouse model

## Abstract

The Calcium-sensing receptor (CaSR) senses extracellular calcium, regulates parathyroid hormone (PTH) secretion, and has additional functions in various organs related to systemic and local calcium and mineral homeostasis. Familial hypocalciuric hypercalcemia type I (FHH1) is caused by heterozygous loss-of-function mutations in the *CaSR* gene, and is characterized by the combination of hypercalcemia, hypocalciuria, normal to elevated PTH, and facultatively hypermagnesemia and mild bone mineralization defects. To date, only heterozygous *Casr* null mice have been available as model for FHH1. Here we present a novel mouse FHH1 model identified in a large ENU-screen that carries an c.2579 T > A (p.Ile859Asn) variant in the *Casr* gene (*Casr*^BCH002^ mice). In order to dissect direct effects of the genetic variant from PTH-dependent effects, we crossed *Casr*^BCH002^ mice with PTH deficient mice. Heterozygous *Casr*^BCH002^ mice were fertile, had normal growth and body weight, were hypercalcemic and hypermagnesemic with inappropriately normal PTH levels and urinary calcium excretion replicating some features of FHH1. Hypercalcemia and hypermagnesemia were independent from PTH and correlated with higher expression of claudin 16 and 19 in kidneys. Likewise, reduced expression of the renal TRPM6 channel in *Casr*^BCH002^ mice was not dependent on PTH. In bone, mutations in *Casr* rescued the bone phenotype observed in *Pth* null mice by increasing osteoclast numbers and improving the columnar pattern of chondrocytes in the growth zone. In summary, *Casr*^BCH002^ mice represent a new model to study FHH1 and our results indicate that only a part of the phenotype is driven by PTH.

**Supplementary Information:**

The online version contains supplementary material available at 10.1007/s00424-024-02927-y.

## Introduction

The Calcium-sensing receptor (CaSR) is a G protein-coupled receptor that senses extracellular concentrations of ionized calcium [14]. The CaSR forms homodimers and is highly expressed in several tissues central to the regulation of systemic calcium homeostasis including parathyroid glands, bone, cartilage, various segments of the gastrointestinal tract, and kidneys. In parathyroid glands, the CaSR regulates the synthesis and secretion of parathyroid hormone (PTH) suppressing PTH release when ionized calcium concentrations are high. In kidney, CaSR is expressed at the basolateral membrane of cells of the thick ascending limb of the loop of Henle (TAL) where it regulates paracellular calcium and magnesium reabsorption mediated by claudin 16 (cdn16) and claudin 19 (cldn19). Under conditions of elevated calcium, the CaSR downregulates clnd16 and 19 in part through enhancing claudin 14 (cldn14) which inhibits cldn16 and cldn19 [14]. These effects of the CaSR are PTH-independent [18, 22]. Stimulation of CaSR activity may also inhibit the Na^+^/K^+^/2Cl^−^ -cotransporter NKCC2 in TAL cells. Also, NCC activity may be regulated by the CaSR [6]. Whether the CaSR is also localized to other nephron segments has remained controversial [13, 22, 25]. In proximal tubule, CaSR may inhibit bicarbonate reabsorption via the NHE3 Na^+^/H^+^-exchanger and phosphate reabsorption by the NaPi-IIa cotransporter [2, 5]. Also, inhibition of AQP2-mediated water reabsorption and stimulation of H^+^-ATPase activity in the collecting duct system has been suggested [24, 27].

Familial hypocalciuric hypercalcemia type I (FHH1, OMIM: #145980) is a genetic disorder characterized by hypercalcemia, hypocalciuria, normal to mildly elevated PTH levels, and in some patients mildly reduced bone mineral density and hypermagnesemia [14]. FHH1 is caused by heterozygous loss-of-function mutations in the gene encoding the CaSR [14, 21]. Heterozygous Casr KO mice have been described as a model of FFH1 but only partially characterized [15].

The *Casr*^BCH002^ mouse line has been identified in a large N-ethyl-N-nitrosourea (ENU)-driven mutagenesis screen searching for bone-related parameters that included alkaline phosphatase, total calcium, and inorganic phosphate [26]. Heterozygous *Casr*^BCH002^ mice had normal alkaline phosphatase but elevated plasma total calcium and lower phosphate. Histological analysis of the line revealed enlarged parathyroid glands and higher median PTH levels. Genetic analysis identified a c.2579 T > A (p.Ile859Asn) variant in the CaSR. This variant, affecting a residue within a transmembrane region of the CaSR [16], is listed in the ClinVar database as a variant of uncertain significance while the SIFT structure tool (https://sift.bii.a-star.edu.sg/) lists it as deleterious. Thus, the structural predictions together with the already known phenotype suggest that *Casr*^BCH002^ mice may be a model for FHH1.

The aims of this study were to examine whether *Casr*^BCH002^ mice are a model of FHH1 and whether some of the phenotypic changes of *Casr*^BCH002^ mice were dependent on PTH. To this end, we examined not only *Casr*^BCH002^ mice but also *Pth* KO and *Casr*^BCH002^/*Pth* KO mice.

## Materials and methods

### Animals

The generation, breeding, and genotyping of PTH deficient mice has been described previously [9, 19]. *Pth* null mice were in a pure C57BL/6 J background. BCH002 mice, harboring a putatively inactivating mutation in the *Casr* gene, were derived from an ENU-based genetic screen [26]. Genetic analysis of the line identified a heterozygous c.2579 T > A, p. Ile859Asn mutation in the *Casr* gene (*Casr*^BCH002^). *Casr*^BCH002^ mice were originally in C3HeB/FeJ background and crossed with *Pth* heterozygous over three generations to yield all desired genotypes with a mixed background. To evaluate the effect of the CaSR on renal function independent from PTH, heterozygous BCH002 and *Pth* null mice were crossed and further bred resulting in 4 different genotypes: wildtype for both genes (WT/WT), heterozygous for the *Casr*^BCH002^ mutation (WT/ *Casr*^BCH002^), homozygous for PTH deficiency (*Pth* KO) (*Pth* KO/WT), and heterozygous for the *Casr* ^BCH002^ mutation and homozygous for *Pth* deficiency (*Pth* KO/ *Casr*^BCH002^). All mice used were littermates and had a mixed C3H-C57 background. Only male mice were used at the age of 8–10 weeks.

All experiments were performed according to Swiss Animal Welfare laws and approved by the local veterinary authority (Kantonales Veterinäramt Zürich) under the numbers ZH156/16 and ZH240/19.

#### Determination of urinary and plasma metabolites

Urinary and plasma levels of total calcium, inorganic phosphate (Pi) and creatinine (enzymatically for plasma) were measured on a UniCel DxC 800 Synchron Clinical System (Beckman Coulter) in the Zurich Integrative Rodent Physiology (ZIRP) facility. Blood levels of ionized calcium were measured with a blood-gas analyzer (Epoc blood gas analysis system, Siemens, Germany). Plasma levels of intact FGF23 (Immutopics; #60–6800), the C-terminal FGF23 fragment (Immutopics; #60–6300) and PTH (Immutopics; #60–2305) were determined by ELISA according to the manufacturer’s protocols.

#### Protein extractions and preparation of brush border membranes (BBM)

For the extraction of total proteins, kidneys were homogenized in RIPA buffer containing 50 mM Tris–HCl (pH 7.4), 150 mM NaCl, 1% NP-40 and 0.5% Deoxycholate acid sodium salt, supplemented with Phenylmethylsulfonyl fluoride (PMSF) and protease inhibitor cocktail (Complete; Roche Diagnostics, Basel, Switzerland). The homogenate was centrifuged at 2000 rpm for 20 min at 4 °C. The resulting supernatant was further centrifuged at 41,000 rpm for 1 h at 4 °C in order to enrich the membrane proteins in the final pellet. Renal BBM were prepared according to the Mg^2+^ precipitation technique [3]. BBM were resuspended in 300 mM Mannitol, 20 mM HEPES-Tris, pH 7.4 and stored at -20 °C. Protein concentrations were determined with the Bio-Rad Protein Assay (Bio-Rad, Hercules, CA).

#### Immunoblotting

The expression levels of transporter proteins were quantified on either total membrane extractions or BBM, while calbindin D28k and Cyp24a1 were determined in total protein extractions. To this end, 10 μg of BBM or 50 μg of either total membrane or total protein were solubilised in Laemmli buffer and separated on SDS-PAGE and transferred to polyvinylidene difluoride membranes (EMD Millipore, Billerica, MA). After blocking nonspecific binding with 5% milk powder in Tris-buffered saline containing 0.1% Tween-20 for 1 h, the blots were incubated overnight at 4 °C with the primary antibodies. Antibodies used were directed against NaPi-IIa (1:2000) [8], NaPi-IIc (1:1000) [20], total NCC (1:2000; a kind gift of J. Loffing [17]), NKCC2 (1:2000, a kind gift of J. Loffing) [31], NHE3 (1:1000; StressMarq; SPC-400D), TRPV5 (1: 500, a kind gift by O. Bonny, [29]), TRPM6 (1:2000, a kind gift by J. Loffing, [28]), the vitamin D receptor (Vdr) (1:500 Santa Cruz), Cyp24a1 (1:1000, Proteintech), the Ca^2+^-sensing receptor (CaSR) (1:500, Thermo Fisher) and calbindin D28k (1:500, Swant). After washing and further blocking, blots were incubated with the appropriate secondary antibodies for 1 h at room temperature. Finally, membranes were exposed to chemiluminescent substrate for 5 min, and protein signals were detected on a LAS-4000 Luminescent Image Analyzer. All images were quantified with Advanced Image Data Analyzer (AIDA; Raytest). The expression of the proteins of interest was normalized to the Ponceau signal.

#### Micro-computer tomography

Right tibias stored at -80 °C were defrosted and scanned with a µCT40 (Scanco Medical, Wangen-Brüttisellen, Switzerland), following the manufacturer’s instructions. Briefly, whole tibia and distal epiphysis were imaged using 20 mm or 5 mm fields of view, respectively, at a tube current of 100 μA, 90 kV tube voltage and 3 min scan time. A 3D Gaussian filter (sigma 0.8, support 1) was applied to reduce the noise present in the images. A 1200 mg/cm^3^ hydroxy-apatite phantom from the company was used to translate greyscales into bone mineral density (BMD). BMD and bone volume data were assessed with the Analyze 12.0 program (Analyze Direct, Inc). We analyzed the bone parameters following previously published guidelines [4].

#### Histomorphometry

After µCT analysis, the right tibia was fixed in 4% paraformaldehyde (PFA), decalcified in 10% EDTA for 3 to 4 weeks and embedded in paraffine. Five µm sections were deparaffinized and washed with PBS for histological analysis. For tartrate-resistant acid phosphatase (TRAP) staining, two slides per animal were incubated in Tris–HCl 0.2 M pH 9.5 for 45 min at 37 °C, and then for 15 min following the TRAP kit instructions (Sigma 387A-1KT). We evaluated the number and/or area of active osteoclasts per total area. Alcian blue safranin (AAS) staining and alkaline phosphatase (ALP) staining were performed as previously described [11, 30]. With the AAS staining we evaluated the growth plate height and the height of the different zones. On the ALP-stained sections the intensity of the staining in triplicates was evaluated by an examiner blinded to the genotype and a score of the staining intensity was given: negative (0), weak (1), moderate (2), and strong (3). Pictures were made using a digital microscope camera (Olympus E‐330 and Olympus BX41; Olympus). Evaluation of the images was performed using the special bone-imaging program Osteo (v. 2013; Bioquant Image Analysis Corporation, Nashville TN, USA).

#### Semi-quantitative real-time PCR

Total RNA was isolated from kidney using a RNeasy kit (Qiagen, Basel, Switzerland). Bone RNA was isolated form one frozen tibia. Prior to freezing, the epiphysis was cut, and bone marrow was flushed out by centrifugation. Frozen bones were crushed in liquid nitrogen using a mortar. Then, RNA was extracted with Trizol (Invitrogen), followed by phenol–chloroform phase separation. RNA purification was done in all cases using the NucleoSpin RNA kit (Macherey–Nagel). RNA was reverse transcribed with TaqMan Reverse Transcription Kit (Applied Biosystems) after which cDNA was amplified by qPCR using Kapa mix (Sigma-Aldrich) with reporter tagged probes together with forward and reverse primers (Supplementary Table 1). Mouse primers and probe were designed using Primer Express (Applied Biosystems, USA) and purchased from Microsynth, Switzerland. Probes were labelled with the reporter dye FAM at the 5ʹ end and the quencher dye TAMRA at the 3ʹ end. The specificity of the primer was tested using adult mouse kidney or tibia cDNA by conventional PCR. Each pair of primer resulted only in a single band of the expected size (data not shown). qPCR reactions were performed on a 7500 Fast Real Time PCR System. Gene expression was normalized to HPRT or GAPDH and the obtained data was analyzed with the 7500 Fast Real-Time PCR System Sequence Detection Software v1.4. The relative gene expression was calculated according to the formula 2^(Ct (control) −Ct (gene of interest))^.

#### Statistics

Statistical analyses were performed using GraphPad Prism version 9.3.1 for Windows (GraphPad Software, San Diego, California USA, http://www.graphpad.com). Statistical significance, defined as *P* < 0.05, was calculated using two-way ANOVA followed by multiple comparisons using the Tukey method. P values are displayed as: * *P* < 0.05, ** *P* < 0.01, *** *P* < 0.001, **** *P* < 0.0001. Data are presented as mean ± SEM.

## Results

### Aberrant mineral balance in Casr^BCH002^ mice is independent from PTH

**Fig. 1:**
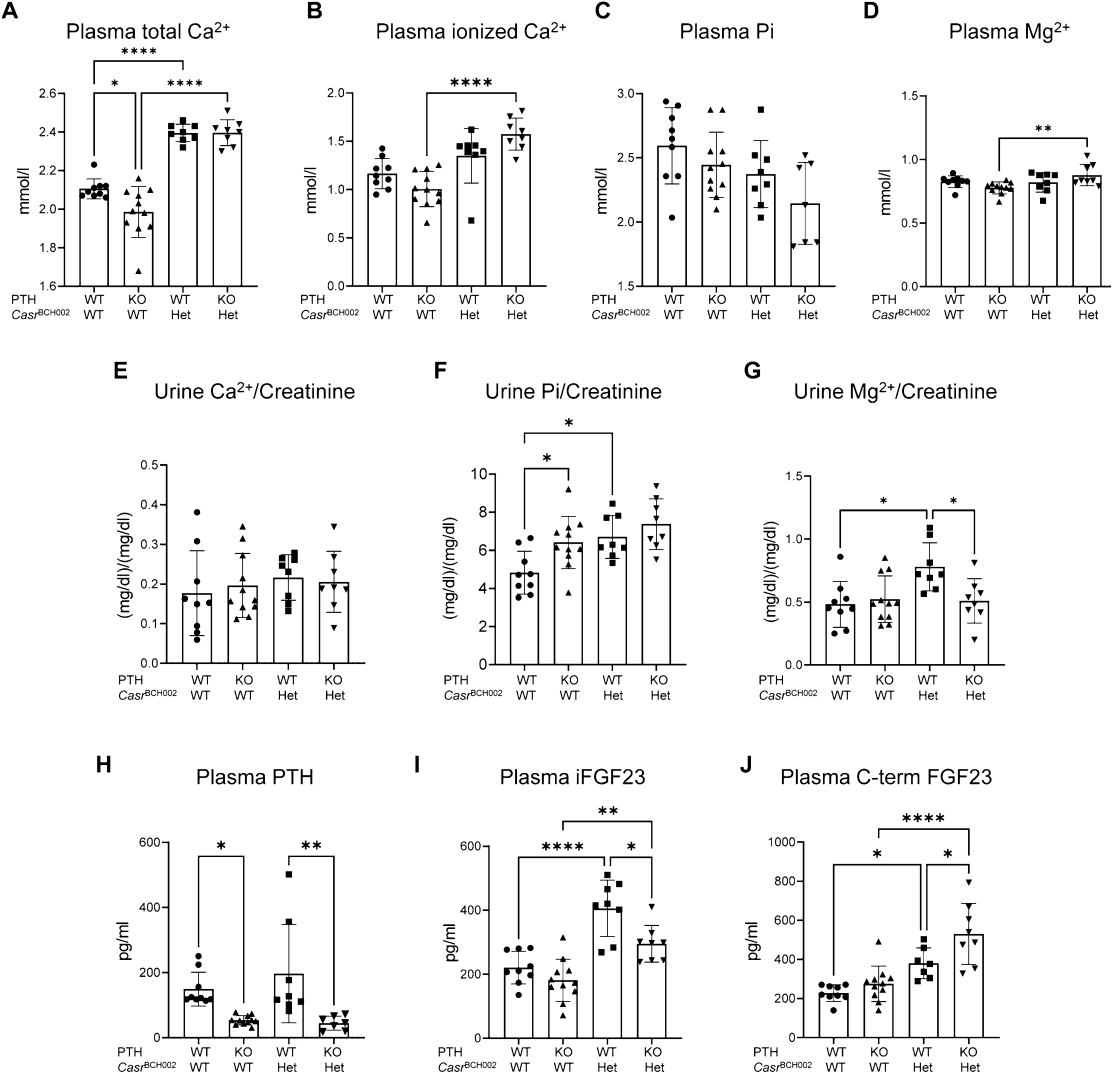
Parameters of mineral metabolism. Plasma and urine analysis for parameters of phosphate and calcium homeostasis in wildtype (WT/WT), *Pth* KO/*Casr*^BCH002^, WT/ *Casr*^BCH002^ and *Pth* KO/*Casr*^BCH002^ mice. Measurements in plasma of (**A**) total calcium, (**B**) ionized Ca^2+^, (**C**) phosphate (Pi), (**D**) magnesium (Mg^2+^). Urinary excretion of (**E**) Ca^2+^, (**F**) phosphate (Pi), and (**G**) Mg^2+^, all normalized to creatinine. Plasma values of (**H**) PTH, (I) intact FGF23 (iFGF23), and (**J**) C-terminal FGF23 (C-term FGF23). All data were analysed in groups of *n* = 7–11 animals. Values are presented with means ± SD together with single values. Data were analysed with 2-way ANOVA with Tukey correction for multiple comparisons between PTH and BCH genotypes. * *p* < 0.05, ** *p* < 0.01, **** *p* < 0.0001

We characterized the mineral metabolism of BCH002 mice carrying the c.2579 T > A, p. Ile859Asn mutation in the *Casr* gene and its dependence on PTH. For that, we analyzed four groups of male littermates: mice wildtype for both genes (WT/WT), mice deficient for PTH (*Pth* KO/WT), mice carrying one mutated allele in the *Casr* gene (WT/ *Casr* ^BCH002^), and mice deficient for PTH and with one mutant *Casr* allele (*Pth* KO/ *Casr* ^BCH002^). Mice carrying the homozygous *Casr*^BCH002^ mutation were not viable (data not shown). No or little differences were observed between the 4 groups regarding body weight, tibia and nose-tail length, food and water intake and urine and feces excretion (Supplementary Fig. 1). Plasma total calcium was elevated in *Casr* mutant mice regardless of the presence or absence of PTH (Fig. 1A). Ionized calcium was higher in *Pth* KO/*Casr* ^BCH002^mice than in *Pth* KO/WT animals (Fig. 1B). Plasma Pi levels showed a trend to lower values in *Pth* KO/*Casr* ^BCH002^ than in *Pth* KO/WT mice (Fig. 1C), while plasma magnesium was slightly elevated in these animals (Fig. 1D). Despite hypercalcemia in *Casr* mutant mice, urinary excretion of calcium was similar between groups, i.e. inappropriately normal in hypercalcemic *Casr* mutant mice (Fig. 1E). Urinary Pi excretion was higher in *Pth* KO/WT and WT/*Casr* ^BCH002^ animals when compared to WT/WT mice, and persisted in *Pth* KO/*Casr* ^BCH002^ mice (Fig. 1F). Urinary magnesium excretion was elevated in WT/*Casr*^BCH002^ mice when compared to WT/WT and *Pth* KO/*Casr* ^BCH002^ mice (Fig. 1G). WT/*Casr*^BCH002^ mice had inappropriately comparable PTH levels to WT/WT while only very low PTH signals were detected in mice lacking PTH possibly due to some cross-reactivity of the PTH ELISA kit used (Fig. 1H). Intact and the C-terminal fragment of FGF23 were both elevated due to *Casr* mutations (Fig. 1I, J), also in the absence of PTH. Thus, mutant *Casr* mice show features of familial hypocalciuric hypercalcemia with evidence for primary hyperparathyroidism.

### Altered renal expression of CaSR targets in Casr^BCH002^ mice

**Fig. 2:**
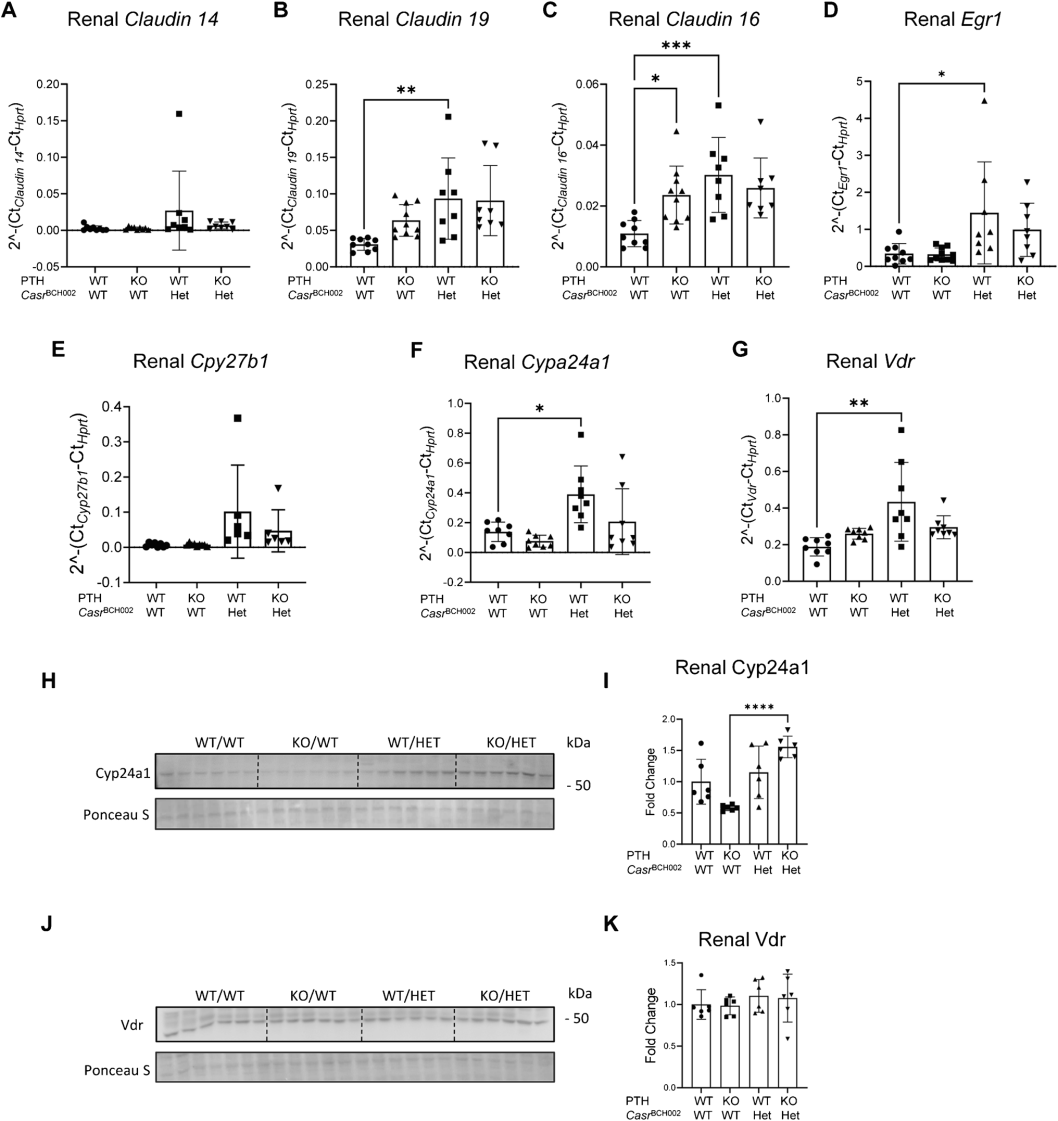
Renal abundance of claudins, Egr1, and calcitriol metabolizing enzymes and vitamin D receptor. mRNA expression and protein abundance was analysed by real time qPCR or immunoblotting in kidneys from wildtype (WT/WT), *Pth* KO/*Casr*^BCH002^ WT, WT/*Casr*^BCH002^ and *Pth* KO/*Casr*^BCH002^ mice. mRNA expression of (**A**) *claudin14*, (**B**) *claudin19*, (**C**) *claudin16*, (**D**) *egr1*, (**E**) *1-α-hydroxylase* (C*yp27b1)*, (**F**) *24-hydroxylase* (C*yp24a1)*, and (**G**) *vitamin D receptor* (*Vdr*) were assessed relative to the housekeeping gene *Hprt*. Protein expression of (**H**, **I**) Cyp24a1 (60 kDa) and (**J**, **K**) Vdr (47 kDa) were normalized to Ponceau S staining. Values are presented as means ± SD (*n* = 8–10) together with single values. Data were analysed with 2-way ANOVA with Tukey correction for multiple comparisons between PTH and BCH genotypes. * *p* < 0.05, ** *p* < 0.01, *** *p* < 0.001, **** *p* < 0.0001

Next, we examined the renal expression of transcripts and proteins regulated by CaSR or involved in renal calcium and Pi handling. Claudin 14 mRNA was similarly low expressed in all four genotypes (Fig. 2A), while claudin 19 and 16 mRNA were higher in WT/*Casr*^BCH002^ animals when compared to WT/WT, with a similar trend in *Pth* KO/*Casr*^BCH002^ mice (Fig. 2B, J). mRNA abundance of Egr1, a target gene of FGF23, was higher in *Casr* mutant mice (Fig. 2D) consistent with elevated FGF23 levels (Fig. 1I, J) in these mice. mRNA expression of Cyp27b1, the enzyme catalyzing the hydroxylation of calcidiol to calcitriol, was similarly low in all groups of mice (Fig. 2E), but Cyp24a1 and the vitamin D receptor (Vdr) transcripts showed higher values in WT/*Casr*^BCH002^ mice compared to WT/WT (Fig. 2F, G). However, protein abundance of Cyp24a1 was elevated only in *Pth* KO/*Casr*^BCH002^ mice as compared to *Pth* KO/WT (Fig. 2H, I), while Vdr protein expression was similar in all groups (Fig. 2J, K).

**Fig. 3:**
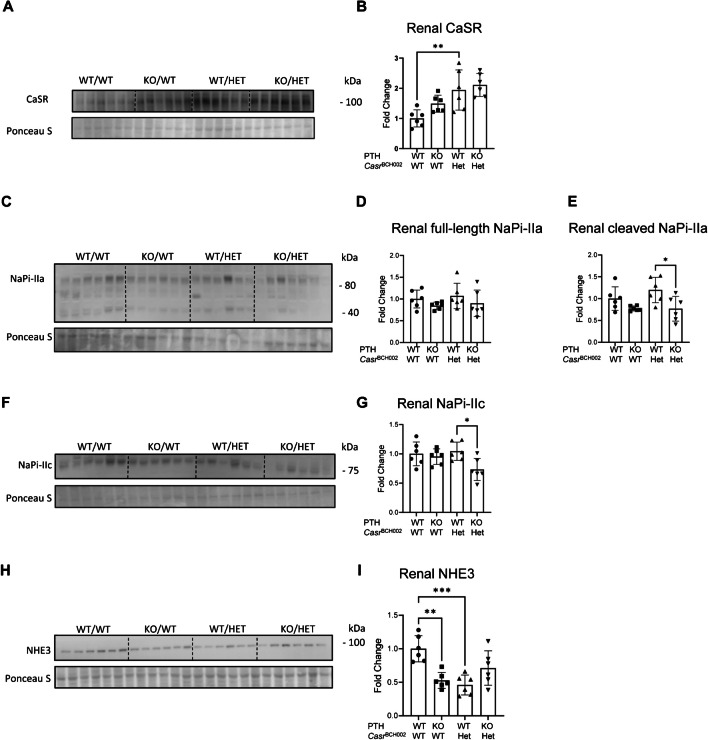
Expression of key proteins involved in renal calcium and phosphate handling. Protein abundance was assessed by immunoblotting in total homogenates (Casr) or brush border membranes (NaPi-IIa, NaPi-IIc and NHE3) from kidneys of wildtype (WT/WT), *Pth* KO/*Casr*^BCH002^, WT/*Casr*^BCH002^ and *Pth* KO/*Casr*^BCH002^ mice. Protein expression of (**A**, **B**) CaSR (130 kDa), (**C**–**E**) NaPi-IIa (full length 85 kDa and cleaved COOH-terminal fragment 45 kDa), (**F**, **G**) NaPi-IIc (80 kDa), and (**H**, **I**) Na^+^/H^+^-exchanger isoform 3 (NHE3, 93 kDa) were normalized to the corresponding ponceau S staining. Values are presented as means ± SD together with single values (*n* = 6). Data were analysed with 2-way ANOVA with Tukey correction for multiple comparisons between PTH and BCH genotypes. * *p* < 0.05, ** *p* < 0.01, *** *p* < 0.001

Renal protein abundance of CaSR was clearly elevated in mutant mice, though it reached statistical significance only in the presence of PTH (Fig. 3A, B). The cleaved product of the Pi cotransporter NaPi-IIa and the NaPi-IIc cotransporter showed a small reduction in *Pth* KO/*Casr*^BCH002^ mice compared to WT/*Casr*^BCH002^ mice (Fig. 3C–G). The Na^+^/H^+^ exchanger NHE3 was reduced in *Casr* mutant mice and in mice lacking PTH, when compared to WT/WT mice (Fig. 3H, I).

**Fig. 4:**
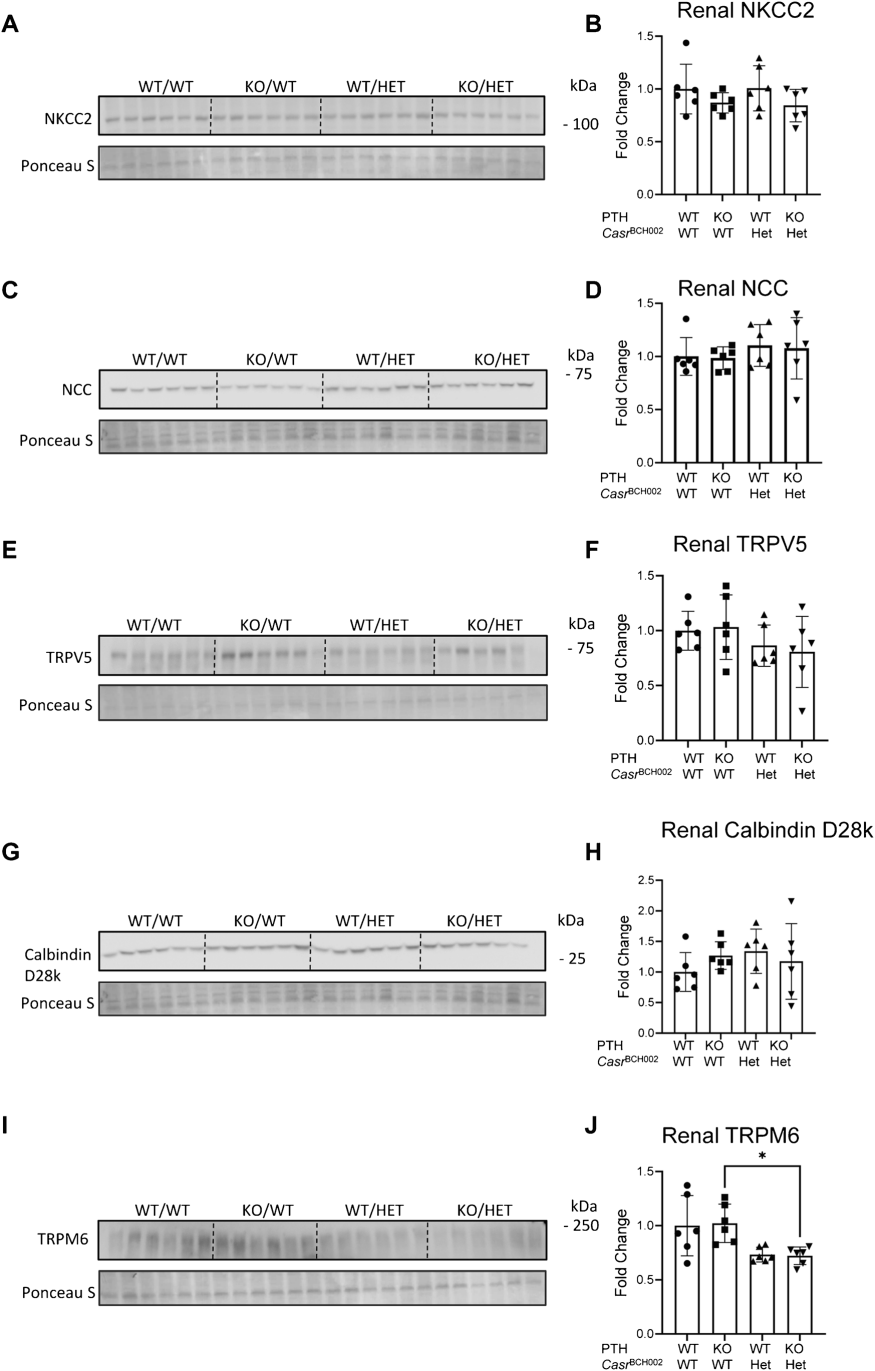
Expression of key proteins involved in renal calcium, magnesium, and salt handling. Protein abundance was assessed by immunoblotting in protein homogenates from kidneys from wildtype (WT/WT), *Pth* KO/*Casr*^BCH002^, WT/*Casr*^BCH002^ and *Pth* KO/*Casr*^BCH002^ mice. Protein expression of (**A**, **B**) NKCC2 (120 kDa), (**C**, **D**) NCC (130 kDa), (**E**, **F**) TRPV5 (75 kDa) (**G**, **H**) Calbindin D28k (28 kDa), and (**I**, **J**) TRPM6 (230 kDa) were normalized to the corresponding ponceau S staining. Values are presented as means ± SD together with single values (*n* = 6/group). Data were analysed with 2-way ANOVA with Tukey correction for multiple comparisons between PTH and BCH genotypes. * *p* < 0.05

Next, we assessed the abundance of several proteins involved in renal salt and calcium handling in the thick ascending limb of the loop of Henle, the distal convoluted tubule and connecting tubule. We found no alterations in the expression of the Na/K/2Cl-cotransporter NKCC2, the thiazide-sensitive NaCl-cotransporter NCC, the calcium channel TRPV5, and the calcium-buffering protein calbindin D28k (Fig. 4A–H). In contrast, the magnesium channel TRPM6 was reduced in *Casr* mutant mice in the absence of PTH (Fig. 4I, J).

### Bone phenotype

**Fig. 5:**
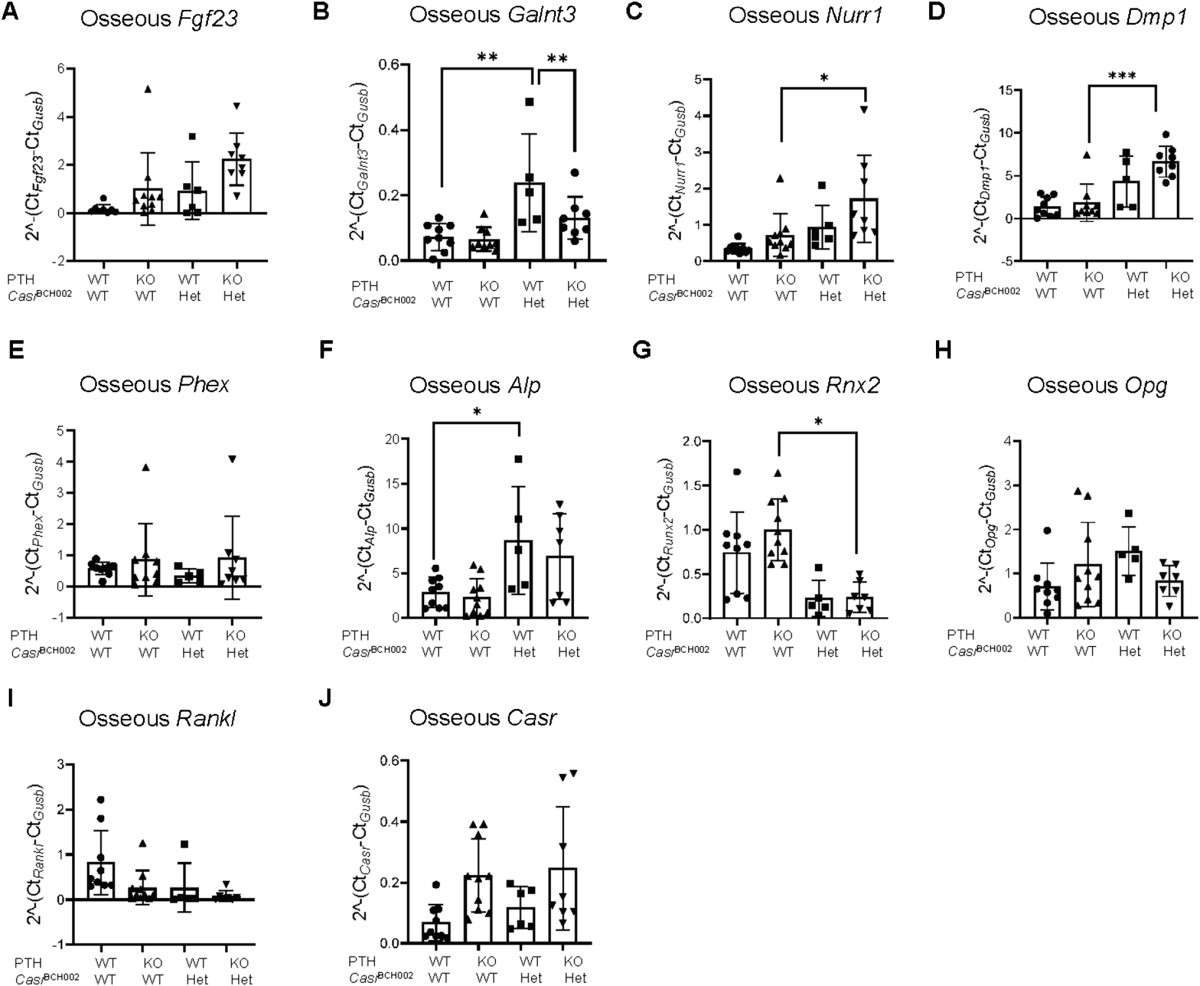
mRNA expression of bone markers in tibia. mRNA expression was analysed by RT-qPCRs in tibias from wildtype (WT/WT), *Pth* KO/*Casr*^BCH002^, *Pth* WT/*Casr*^BCH002^ and *Pth* KO/*Casr*^BCH002^ mice. (**A**) *Fgf23*, (**B**) *Galnt3*, (**C**) *Nurr-1*, (**D**) *Dmp1*, (**E**) *Phex*, (**F**) *Alp*, (**G**) *Rnx2,* (**H**) *Opg*, (**I**) *Rankl*, (**J**) *Casr* relative to the housekeeping gene *Gusb.* Values are presented as means ± SD together with single values (*n* = 8–10). Data were analysed with 2-way ANOVA with Tukey correction for multiple comparisons between PTH and BCH genotypes. * *p* < 0.05, ** *p* < 0.01, *** *p* < 0.001

In a last series we assessed bone in the four groups of mice. We first analyzed the mRNA abundance of various bone markers and factors involved in FGF23 synthesis. Higher circulating levels of intact FGF23 in *Casr* mutant mice did not correlate with increased *Fgf23* gene expression, but correlated with higher mRNA abundance of *Galnt3*, the enzyme stabilizing FGF23 through O-Glycosylation, in the WT/*Casr*^BCH002^ group (Fig. 5A, B). Small differences in mRNA abundance were found for *Nurr1*, a transcription factor involved in FGF23 regulation, and *Dmp1*, a negative regulator of FGF23. Both were elevated in *Pth* KO/*Casr*^BCH002^ mice compared to *Pth* KO/WT suggesting an effect of mutant Casr (Fig. 5C, D). Phex, another negative regulator of FGF23 showed no differences between the groups (Fig. 5E). We also found an increase in *Alp*, alkaline phosphatase, a marker of osteoblasts (Fig. 5F), in WT/*Casr*^BCH002^ mice and a decrease in *Rnx2*, a marker of osteoclasts, in WT/*Casr*^BCH002^ (non-significant) and *Pth* KO/*Casr*^BCH002^ (significant) mice suggesting a change in the balance between osteoblasts and osteoclasts (Fig. 5G). No differences were found between the groups in *Opg* and *Rankl* mRNA abundance, markers of bone formation and resorption (Fig. 5H, I). Also no differences were observed in the mRNA abundance of CaSR (Fig. 5J).

**Fig. 6:**
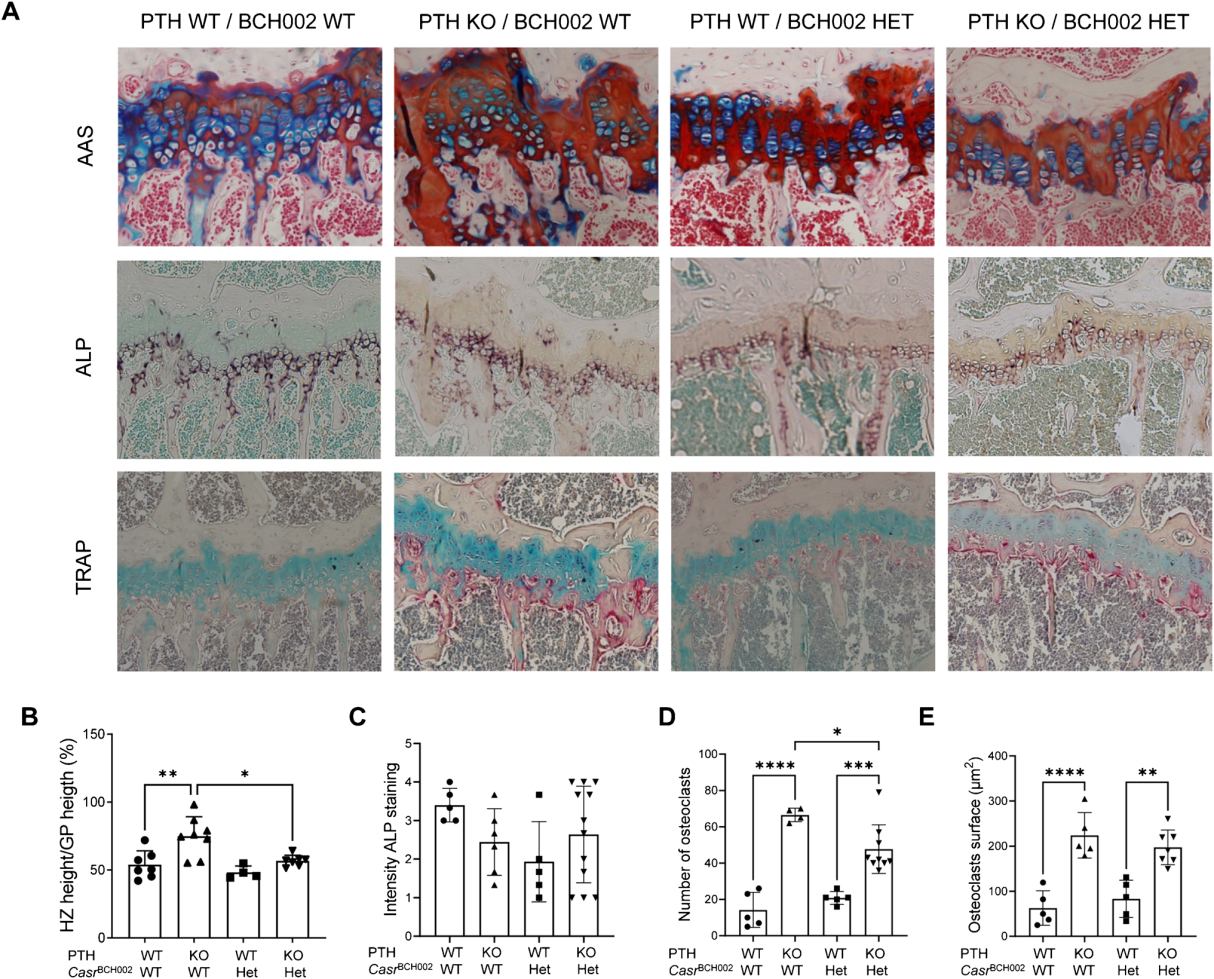
Analysis of tibia growth plate. The growth plate of tibias from wildtype (WT/WT), *Pth* KO/*Casr*^BCH002^, WT/*Casr*^BCH002^ and *Pth* KO/*Casr*^BCH002^ mice was analyzed using immunohistochemistry. (**A**) Upper panels: Alcian blue safranin (AAS) staining for mature cartilage (red) and connective tissue (blue):; middle panels: alkaline phosphatase (ALP, purple) to identify osteoblasts and counterstain of tissue (green), and lower panels: Tartrate-resistant alkaline phosphate (TRAP) to identify osteoclasts (pink). Blue staining for connective tissue and nuclei stained in purple. Morphometric analysis of (**B**) the ratio between the hypertrophic zone (HZ) and growth plate (GP), (**C**) the intensity of ALP staining, (**D**) number of osteoclasts, and (**E**) the surface covered by osteoclasts. Values are presented as means ± SD (*n* = 5). Data were analysed with 2-way ANOVA with Tukey correction for multiple comparisons between PTH and BCH genotypes. * *p* < 0.05, ** *p* < 0.01, *** *p* < 0.001, **** *p* < 0.0001

Histomorphology was used to analyze the growth plate of tibias (Fig. 6A). Absence of PTH caused a profound disturbance of the columnar pattern of chondrocytes in the growth zone which was less visible in *Casr* mutant animals (WT/*Casr*^BCH002^), and this disturbance was even partially restored by the *Casr* mutation in *Pth* KO/*Casr*^BCH002^ mice (Fig. 6A). Similarly, the increase in the ratio of hypertrophic to growth plate height zone associated with PTH depletion was rescued in *Pth* KO/*Casr*^BCH002^ mice when compared to *Pth* KO/WT mice (Fig. 6B). ALP positive was present only in the deep layers of articular cartilage adjacent to the subchondral bone. No differences were observed in the intensity and distribution of osteoblasts (Fig. 6C). The number of osteoclasts was highly increased in the absence of PTH and this increase was partly blunted by the *Casr* mutant animals (Fig. 6D). The *Casr* mutation had no effect on osteoclasts surface neither in the presence nor in the absence of PTH (Fig. 6E).

We further analyzed bone by μCT. Despite the changes in bone morphology, no major alterations were observed by μCT analysis in bone between the groups (Supplementary Table 2).

## Discussion

In this study we present a new mouse model for familial hypocalciuric hypercalcemia (FHH1) due to an inactivating *Casr* mutation and analyze to which extent the phenotype is dependent on PTH. Major signs of FHH1 include hypercalcemia, hypocalciuria, normal to elevated PTH, and variably hypophosphatemia, hypermagnesemia, and mild bone mineralization defects. We thus focused our examinations on these aspects. FFH is a relatively benign disorder due to dominant loss-of-function variants in the *Casr* gene [14, 21]. The *Casr*^BCH002^ mice were identified in a large ENU-mutagenesis screen based on hypercalcemia and hypophosphatemia [26]. However, the latter was only present in half of the animals. Mutant mice had been reported to have also slightly elevated PTH levels and enlarged parathyroid glands [26]. The mutation was mapped to the *Casr* locus and a c.2579 T > A, (p. Ile859Asn) variant detected. This mutation is located in the transmembrane region of the Casr protein and is predicted to impair function of the CaSR. Whether the mutation exerts a dominant negative effect or the phenotype of mice is due to haploinsufficiency of the *Casr* gene remains unclear at this point. The initial mouse line had already been crossed back for more than 15 generations likely diluting possible other bystander variants caused by ENU [26]. We crossed the line further over at least three more generations into *Pth* KO mice. Of note, the initial line had been in the C3HeB/FeJ background while *Pth* KO mice were in a C57BL/6 J background. Thus, our mouse line is in a mixed background and we strictly used only littermates for experiments. The changes in genetic background and standard rodent chow used in our facility may also explain some minor differences in phenotype between the initial description of the BC002 line and our findings discussed below.

*Casr*^BCH002^ mice recapitulate several features of FHH1 including hypercalcemia, an inappropriately normal PTH which is higher than expected for the degree of hypercalcemia. We also found inappropriately normal urinary calcium excretion which is lower than expected for hypercalcemia, and hypermagnesemia. It has been suggested that elevated FGF23 levels may distinguish FHH1 from other forms of PTH-dependent hypocalciuric hypercalcemia [1]. Our mouse model showed higher intact FGF23 levels and this was persistent in the absence of PTH. Likewise, the C-terminal fragment of FGF23 is higher in *Casr*^BCH002^ mutant animals with and without PTH. In WT/*Casr*^BCH002^ mice we also found higher *Galnt3* mRNA in bone which serves to stabilize FGF23. In mice lacking both the Casr and PTH manipulations of calcium and phosphate caused increases in FGF23 only when the calcium-phosphate product (Ca x Pi) exceeded a threshold [23]. The mechanism sensing the Ca x Pi remains elusive.

The CaSR regulates PTH secretion by the parathyroid glands. Reduced activity of the CaSR shifts the relationship between ionized calcium and PTH to the right (i.e., suppression of PTH release requires higher calcium levels) as also apparent in the Casr^BCH002^ mice. Since PTH is a major hormone regulating calcium, phosphate, and bone metabolism, we asked whether some of the features of FHH1 might be dependent on PTH. Several findings suggest that part of the phenotype is independent from PTH, namely hypercalcemia, and higher FGF23 (both intact and C-terminal fragment). In kidney, the transcriptional upregulation of cldn16 and cldn19 as well as the downregulation of NHE3 and TRPM6 protein expression are independent from PTH. Moreover, upregulation of Galnt3 and downregulation of Rnx2 transcripts in bone are independent of PTH. In the sole absence of PTH, osteoclast numbers were increased while in *Casr*^BCH002^ mutant mice without PTH, this increase was partly blunted. Thus, several of the key features of FHH1 are unrelated to PTH but appear to be caused by mutant *Casr*. Whether all these phenotypes are directly linked to mutant *Casr* or indirectly, e.g. via elevated FGF23 (e.g. phosphaturia) or hypermagnesemia (e.g. lower TRPM6 levels) remains to be established. Our data are also consistent with findings in rats demonstrating a PTH-independent regulation of renal calcium handling by the CaSR [18] which may contribute also to the phenotype observed in *Casr*^BCH002^ mice.

Previously, homo- and heterozygous *Casr* KO mice have been generated. While the full ablation of the *Casr* mimics neonatal severe hyperparathyroidism, heterozygous mice have been suggested as model for FHH1 [15]. These heterozygous mice have higher total and ionized blood calcium with elevated PTH levels. Moreover, serum magnesium was higher. Urinary calcium excretion in spot urine appeared to be reduced. Our *Casr*^BCH002^ mice recapitulate most of these findings except for PTH, however, Ho et al. had measured PTH after an overnight fasting while our mice were fed a libitum. Of note, the phenotype in our mice is less severe than in mice totally lacking Casr which might argue for either only a milder effect of the specific Ile859 Asn mutation with no dominant negative effect or for haploinsufficiency. The latter would be compatible with electrolyte disorders similar to the heterozygous mice. Heterologous expression studies of the mutant receptor may be needed to address this question. Unfortunately, no information is available as to the further phenotype of heterozygous Casr null mice, while our data demonstrate distinct effects in bone and kidney.

It has been debated whether the CaSR is expressed in the renal proximal tubule [5, 18, 25] and it has been suggested that the CaSR may stimulate bicarbonate absorption in the proximal tubule involving the Na^+^/H^+^-exchanger NHE3 [5]. In the *Casr*^BCH002^ mice we found a downregulation of NHE3 which might be explained by the absence of normal CaSR signaling. However, also in *Pth* KO mice and in the *Pth* KO/ *Casr*^BCH002^, NHE3 was downregulated. PTH has been shown to increase internalization and downregulation of NHE3 [7, 12] and that downregulation of NHE3 reduced proximal tubule calcium reabsorption [10]. Thus, the mechanism leading to downregulation of NHE3 in the absence of PTH remains to be established.

In bone, we found no major effect of the *Casr*^BCH002^ mutation except on Alp and Rnx2 mRNA expression. The differences in Alp mRNA, however, did not translate into changes in ALP staining in bone sections. The decrease in Rnx2 in animals with mutant CaSR might suggest a problem in osteoblast formation or differentiation. However, no differences in ALP staining suggest similar osteoblast numbers. In contrast, TRAP stainings suggest that the absence of PTH is associated with higher osteoclast numbers which is surprising as PTH is a major stimulus for the formation of osteoclasts. MicroCT analysis of femurs showed a small reduction in trabecular bone volume in mice lacking PTH (irrespective of the CaSR status) which may confirm the histological data. However, it remains unclear what causes the increase in osteoclast numbers in animals lacking PTH:

In summary, *Casr*^BCH002^ recapitulate major features of patients with FHH1. However, the analysis of double transgenic mice lacking also PTH, suggests that part of the syndrome are rather caused by direct effects of mutant *Casr* than by dysregulated PTH.

### Supplementary Information

Below is the link to the electronic supplementary material.

Supplementary file1 Supplementary Figure 1 Morphometric data, intake and excretion of animals. (A) Body weight (g), (B) nose to tail length (cm), (C) tibia length (cm), (D) 24 hrs food intake normalized to body weight (BW) g/g, (E) 24 hrs water intake normalized to body weight (BW) g/g, (F) 24 hrs urine excretion normalized to body weight (BW) ml/g, and (G) 24 hrs faeces excretion normalized to body weight (BW) g/g. Data were analysed with 2-way ANOVA with Tukey correction for multiple comparisons between PTH and BCH genotypes. * p < 0.05, ** p < 0.01. Supplementary Table 1 Sequences of primers and probes used for RT-qPCR. Supplementary Table 2 MicroCT analysis data. (PDF 1790 KB)

## Data Availability

No datasets were generated or analysed during the current study.
