# Supplementary material for: A novel mouse model for familial hypocalciuric hypercalcemia (FHH1) reveals PTH-dependent and independent CaSR defects
Source: Pflugers Arch. 2024 Feb 22;476(5):833–45. doi: 10.1007/s00424-024-02927-y (PMC11033242; doi:10.1007/s00424-024-02927-y)

Supplementary Figure 1

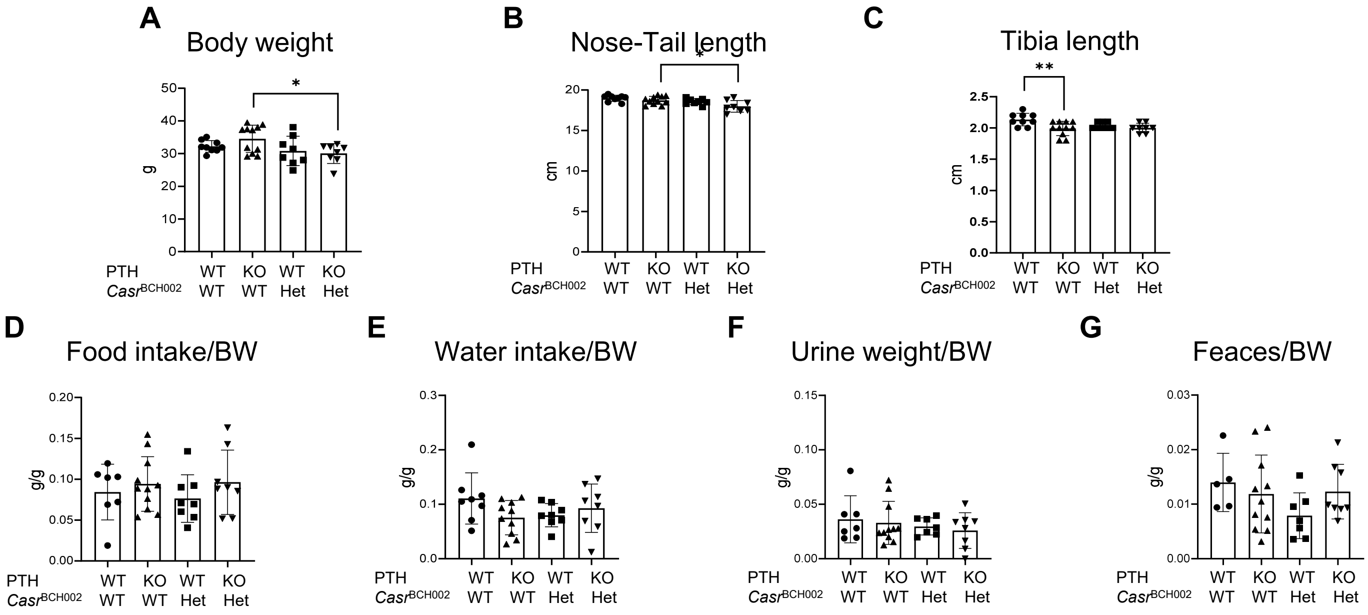

Supplementary Table 1

Gene	Forward Primer / Reverse Primer / Probe
Claudin14	5’-TGT CCT GGA CCA CGA ATG AC-3’
	5’-TGA GGA GAT GAA GCC CAG GT-3’
	SYBR GREEN
Claudin19	5’-TGG ACG GTC ATA TCC AGT CA-3’
	5’-CTT GGC AGT GGG GTT ACT GT-3’
	5’-AGT GTC GTG GGC ATG AAG TGC ACT C-3’
Claudin16	5’-CCT GGA CAG ACT GTT GGA TGG T-3’
	5’-CAG GTT CGA ATC CCA TCA AA-3’
	5’-ATG ACT CCC TGG AGG TGA GCA-3’
Cyp24a1	5’-CCAGCGGCTAGAGATCAAAC-3’
	5’-CACGGGCTTCATGAGTTTCT-3’
	5’-TACGGGCTGATGATCCTGGAAGGAC-3’
Cyp27b1	5’-CCGACCCTACACTTGTGGAG-3’
	5’-ATTCTTCACCATCCGCCGTT-3
	5’-TCTCATCATGGGCAGAGCACCGTC-3’
Vdr	5’-GGA TCT GTG GAG TGT GTG GA-3’
	5’-CAT GCT CCG CCT GAA GAA AC-3’
	5’-TAT GAC CTG TGA AGG CTG CA-3’
Fgf23	5’-GTA TGG ATC TCC ACG GCA AC-3’
	5’-AGT GAT GCT TCT GCG ACA AGT-3’
	5’-TTT TTG GAT CGC TTC ACT TCA GCC C-3’
Nurr-1	5’-CAT CGA CAT TTC TGC CTT CTC-3’
	5’-CTT CCA CTC TCT TGG GTT CCT-3’
	5’-TGC CCT GGC TAT GGT CAC AGA GAG A-3’
Phex	5’-GCT GCC AGA GAA CAA GTG C-3’
	5’-TCC TCA GCT ATG CCA GGA AG-3’
	5’-CAG TCC TCC ACA ATT TAG GGT CAA TGG TG-3’
Galnt3	5’-GAG AAA GAG CGA GGG GAA AC-3’
	5’-GTG GAC CAT GCT TCA TTG TG-3’
	5’-ACA CCC GAC CAC CTG AAT GTA TTG AAC-3’
Dmp1	5’-TCT CCC AGT TGC CAG ATA CC-3’
	5’-TCT CCA GAT TCA CTG CTG TCC-3’
	5’-CTC TGA AGA GAG GAC GGG TGA TTT GG-3’
Casr	5’-AGC CAA CCT GCT GGG ACT T-3’
	5’-CTT ATT GCT GAG AAG CCT GCT AGA-3’
	5’-TCT ATA TCC CCC AGG TGA GCT ACG CCT C-3’
Alp	5’-CCA GAA AGA CAC CTT GAC TGT GG-3’
	5’-TCT TGT CCG TFT CGC TCA CCA T-3’
	SYBR GREEN
Opg	5’-TAC CTG GAG ATC GAA TTC TGC TT-3’
	5’-CCA TCT GGA CAT TTT TTG CAA A-3’
	5’-ACC GGA GCT GTC CCC CGG G-3’
Rankl	5’-CCT GAG GCC CAG CCA TTT-3’
	5’-CTT GGC CCA GCC TCG AT-3’
	5’-CAC CTC ACC ATC AAT GCT GCC AGC-3’
Runx2	5’-CCT GAA CTC TGC ACC AAG TCC T-3’
	5’-TCA TCT GGC TCA GAT AGG AGG G-3’
	SYBR GREEN
Egr1	5’-CAT CGA CAT TTC TGC CTT CTC-3’
	5’-CTT CCA CTC TCT TGG GTT CCT-3’
	5’-TGC CCT GGC TAT GGT CAC AGA GAG A-3’
Hprt	5’-TTA TCA GAC TGA AGA GCT ACT GAA TGA TC-3’
	5’-TTA CCA GTG GTC AAT TGT GTC TTC AAC AAT C-3’
	5’-TGA GAG ATC ATC TCC ACC AAT AAC TTT TAT GTC-3’
Gusb	5’-CTC ATC TGG AAT TTC GCC GA-3’
	5’-GGC GAG TGA AGA TCC CCT TC-3’
	5’-CGA ACC AGT CAC CGC TGA GAG TAA TCG-3’

Supplementary Table 2. Bone parameters assessed by µCT analysis.

Parameter	PTH WT / BCH002 WT n=8	PTH KO / BCH002 WT n=6	PTH WT / BCH002 Het n=9	PTH KO / BCH002 Het n=6	P-value PTH	P-value BCH0002
Bone mineral density (mg/cc)	6779 ± 121	7011 ± 105	6848 ± 178	6895 ± 142	0.3655	0.8743
Cortical volume (mm^3)	2.40 ± 0.13	1.83 ± 0.16*	2.21 ± 0.13	1.82 ± 0.05	0.0012	0.4413
Trabecular volume (mm^3)	1.29 ± 0.15	0.70 ± 0.14	1.23 ± 0.18	0.93 ± 0.10	0.0094	0.5950
Tissue volume (mm^3)	7.61 ± 0.27	7.91 ± 0.30	6.67 ± 0.72	7.21 ± 0.36	0.0591	0.3161
Bone volume (mm^3)	3.29 ± 0.19	2.54 ± 0.28	3.44 ± 0.29	2.75 ± 0.16	0.0090	0.4845
Bone surface (mm^2)	73.45 ± 3.27	57.51 ± 7.42	75.41 ± 5.42	68.02 ± 3.58	0.0340	0.2417
Bone volume/tissue volume	43.10 ± 1.67	38.11 ± 1.42	42.98 ± 2.44	38.09 ± 0.92	0.0183	0.9728
Bone surface/Bone volume (mm^2/mm^3)	22.89 ± 1.71	22.32 ± 1.42	22.30 ± 0.97	24.83 ± 0.74	0.4664	0.4747
Cortical porosity	0.36 ± 0.05	0.43 ± 0.1	0.47 ± 0.05	0.58 ± 0.12	0.2482	0.1053
Pore number	311 ± 43	484 ± 61	470 ± 68	613 ± 107	0.0409	0.0618
Trabecular thickness (mm)	0.087 ± 0.003	0.076 ± 0.003	0.084 ± 0.004	0.085 ± 0.006	0.1686	0.4650
Connectivity density ($1/mm^3$)	107.50 ± 9.88	64.36 ± 11.96	98.50 ± 15.59	85.88 ± 5.74	0.0374	0.6259
Trabecular bone mineral density (mg/cc)	3585 ± 102	3370 ± 147	3567 ± 84	3628 ± 57	0.4571	0.2507

*P-value = 0.05 PTH-WT / $\textit{Casr}^{BCH002}$ -WT vs PTH KO / $\textit{Casr}^{BCH002}$-WT

Supplementary Figure 2

Original blot: Cyp24a1 (Fig 2H)

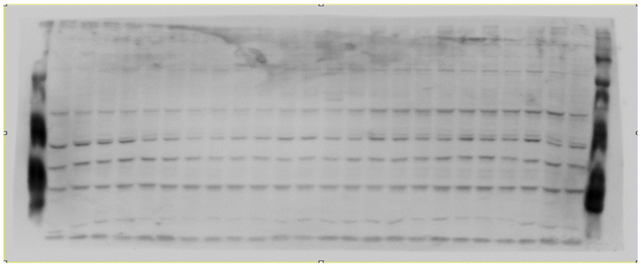

Original blot: Vdr (Fig 2J)

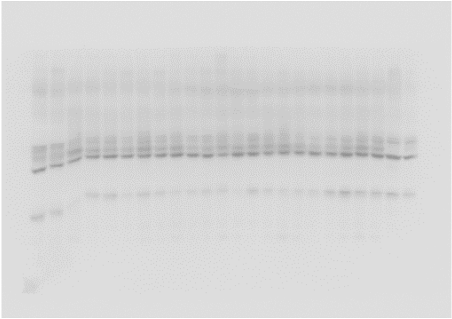

Original blot: Ponceau (Fig 2H)

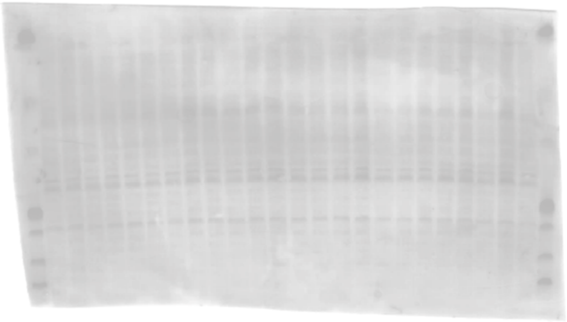

Original blot: Ponceau (Fig 2J)

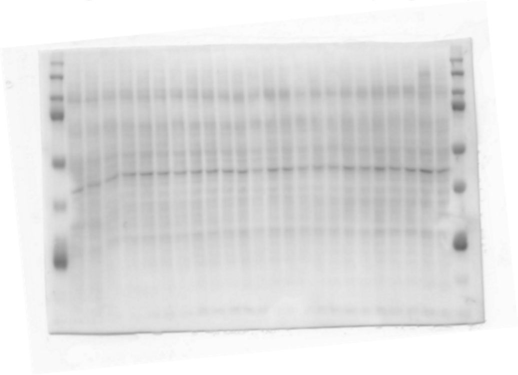

Supplementary Figure 3

Original blot: Casr (Fig 3A)

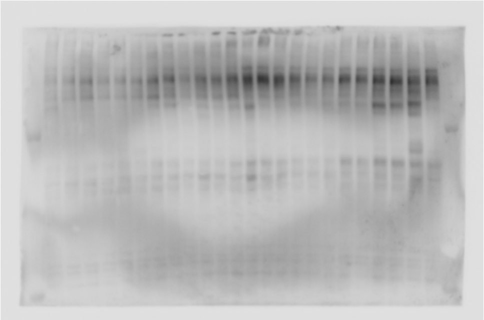

Original blot: Ponceau (Fig 3A)

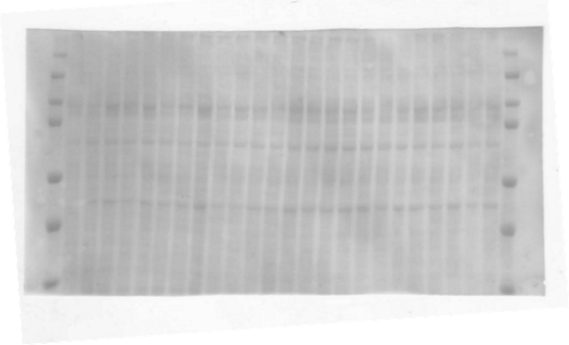

Original blot: NaPi-IIa (Fig 3C)

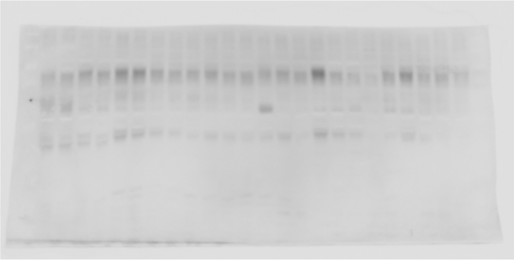

Original blot: Ponceau (Fig 3C)

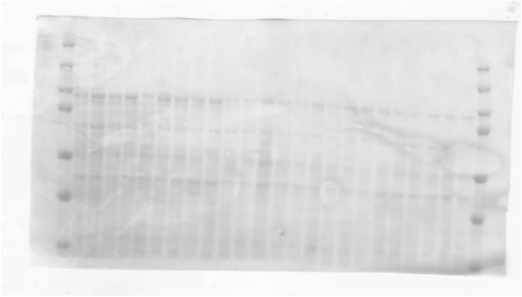

Original blot: NaPi-IIc (Fig 3F)

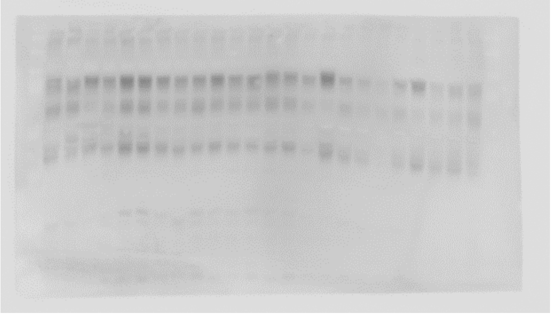

Original blot: Ponceau (Fig 3C)

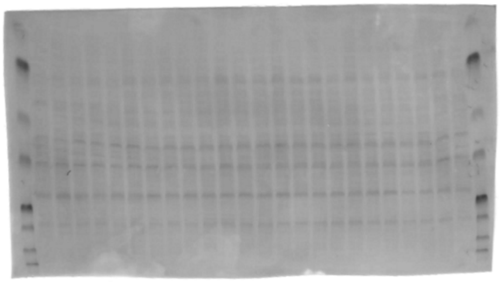

Original blot: NHE3 (Fig 3H)

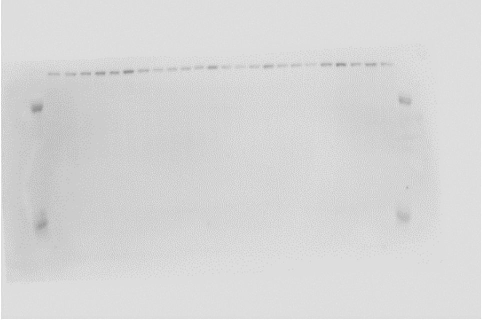

Original blot: Ponceau (Fig 3H)

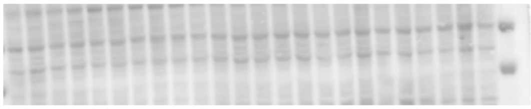

Supplementary Figure 4

Original blot: NKCC2 (Fig 4A)

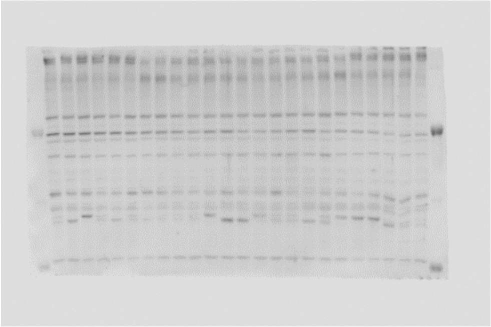

Original blot: Ponceau (Fig 4A)

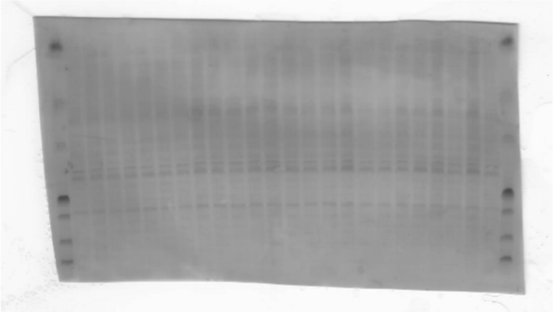

Original blot: NCC (Fig 4C)

Original blot: Ponceau (Fig 4C)

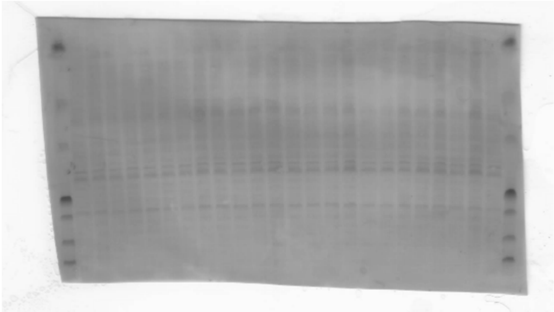

Original blot: TRPV5 (Fig 4E)

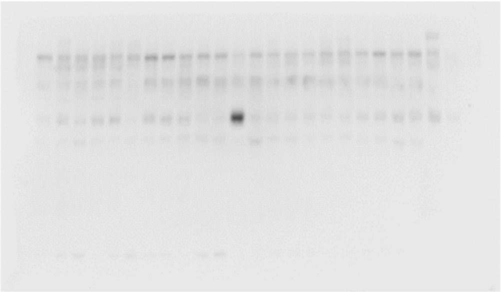

Original blot: Ponceau (Fig 4E)

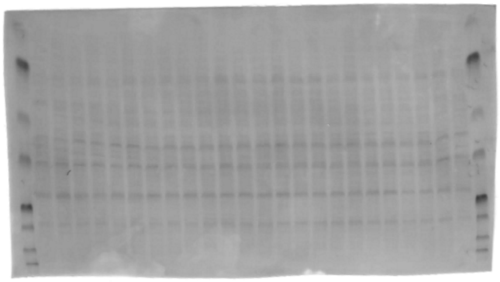

Original blot: calbindin D28k (Fig 4G)

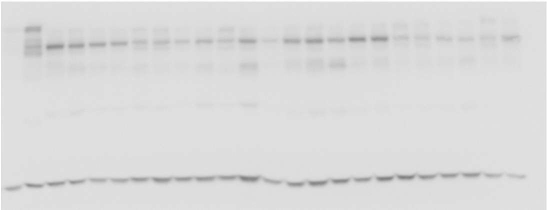

Original blot: Ponceau (Fig 4C)

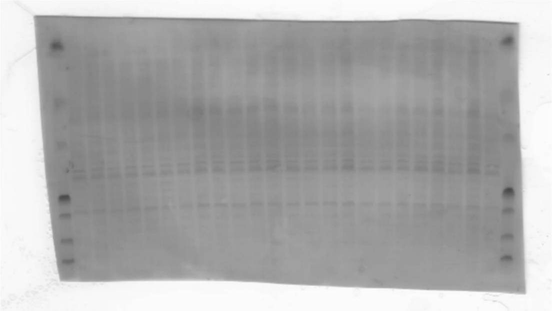

Original blot: TRPM6 (Fig 4I)

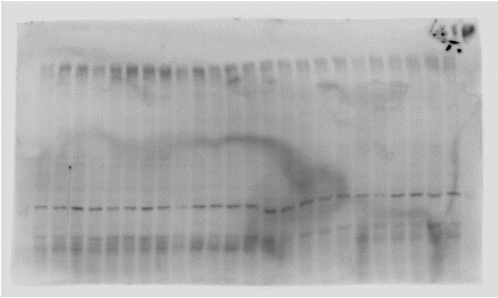

Original blot: Ponceau (Fig 4I)

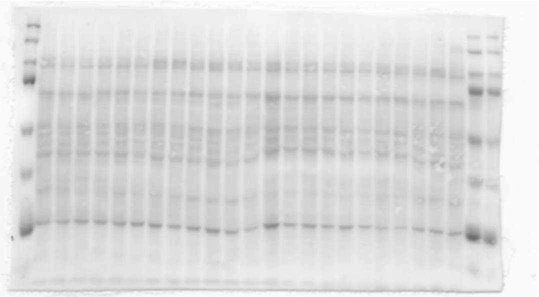

Supplement: Supplementary file 1 — Supplementary file1 Supplementary Figure 1 Morphometric data, intake and excretion of animals. (A) Body weight (g), (B) nose to tail length (cm), (C) tibia length (cm), (D) 24 hrs food intake normalized to body weight (BW) g/g, (E) 24 hrs water intake normalized to body weight (BW) g/g, (F) 24 hrs urine excretion normalized to body weight (BW) ml/g, and (G) 24 hrs faeces excretion normalized to body weight (BW) g/g. Data were analysed with 2-way ANOVA with Tukey correction for multiple comparisons between PTH and BCH genotypes. * p < 0.05, ** p < 0.01. Supplementary Table 1 Sequences of primers and probes used for RT-qPCR. Supplementary Table 2 MicroCT analysis data. (PDF 1790 KB) [file 424_2024_2927_MOESM1_ESM.pdf]
